# Coevolution of exploiter specialization and victim mimicry can be cyclic and saltational

**Published:** 2007-01-11

**Authors:** Niclas Norrström, Wayne M. Getz, Noél M.A. Holmgren

**Affiliations:** 1 School of Life Sciences, University of Skövde, P.O. Box 408, SE-541 28 Skövde, Sweden;; 2 Department of Environmental Sciences, Policy and Management, University of California at Berkeley, Berkeley, CA 94720-3112, USA

**Keywords:** Trophic interaction, speciation, selection, model, perception

## Abstract

Darwin’s Principle of Divergence explains sympatric speciation as gradual and directional. Contradicting evidence suggests that species’ traits evolve saltationally. Here, we model coevolution in exploiter-victim systems. Victims (resource population) have heritable, mutable cue phenotypes with different levels of defense. Exploiters have heritable, mutable perceptual phenotypes. Our simulations reveal coevolution of victim mimicry and exploiter specialization in a saltational and reversible cycle. Evolution is gradual and directional only in the specialization phase of the cycle thereby implying that specialization itself is saltational in such systems. Once linked to assortative mating, exploiter specialization provides conditions for speciation.

## Introduction

Evolutionary biologists debate the directionality of evolving specialization and its role in sympatric speciation (Berlocher and Feder 2002; Mayr 1994). This debate is rooted in Darwin’s Principle of Divergence (Darwin 1859), which states that the most extreme variants of one species tend to take a new promising position in the economy of nature and thereby tend to have more descendants than intermediate variants. If the principle holds, the intermediate types disappear and the original group finally split from each other and speciate. Thus the principle invokes that specialists on the edges of populations separate into distinct subpopulations with the centre collapsing. The specialization process was described by Darwin as being gradual, but contradicting evidence suggests that it is saltational (Gould 2002).

Although recent models support Darwin’s principle (Dieckmann and Doebeli 1999), it has been criticized (Mayr 1994), and a question remains regarding the extent to which reversals in specialization occur, thereby thwarting incipient speciation, particularly under sympatric or heteropatric (i.e. sympatry on heterogeneous landscapes, Getz and Kaitala 1989) conditions. Many specializations in nature are the result of coevolution between two or more species (Ehrlich and Raven 1964). It is unclear whether the coevolution of traits in host-parasitoid, host-disease, prey-predator, or other exploiter-victim systems is generally symmetrical or asymmetrical in nature. In the ecological and evolutionary interactions between phytophagous insects and plants (Jermy and Szentesi 2003; Mcevoy 2002), it is known that plants influence the evolution of the insects, but it is less clear how much the insects affect the plants. Supporters of the symmetrical view believe that insects affect the population dynamics and evolution of plants while their opponents claim that the impact of the insects is weak or non-existent. This debate generalizes to the exploiter-victim systems considered here.

Mimicry is a remarkable example of coevolution (for a review on mimicry see Pasteur 1982), and one of the first strong evidence for Darwin’s theory of evolution (Bates 1862). Mimicry has been observed in species systems as different as fishes (Sazima 2002), spiders (Allan et al 2002), birds (Avilés and Møller 2004) and butterflies (Ehrlich and Raven 1964). Grim (2005) argues that mimicry has been used indiscriminately and inconsistently, and that the mimicry concept should reflect the underlying process. Mimicry is an adaptation evolved by selection pressure from signal receivers and is best understood as a coevolutionary process (Holmgren and Enquist 1999, Grim 2005). This process has been confirmed in computer simulations that use artificial neural nets as models for perceptual systems (Holmgren and Enquist 1999), as well as a study of a parasitic Cuckoo system that includes a decision rule (Servedio and Lande 2003).

## Model

We study coevolutionary dynamics in a computational, ecologically-based population model of exploiter-victim interactions. This model involves exploiters choosing among palatable, intermediate, and unpalatable victims and the presence of Batesian mimicry among victims (the most palatable are selected to look like the least palatable, Clarke and Sheppard 1960). The evolution of exploiter traits is driven by victim traits and vice-versa. Both exploiters and victims are modeled as clonally reproducing populations. The victims have a mutable cue phenotype and, in this first analysis, a fixed defensive phenotype. The fitness of victims is reduced through exploitation, competition within defense phenotype clusters and defense against exploitation, while the fitness of exploiters decreases with both the level of defense among the victims they select and through competition among exploiters for victims. The exploitation rate of a group of victims with a particular cue and defense phenotype is then the number of exploiters weighted by the average preference of the exploiters for victims of that phenotype, divided by the number of victims of that phenotype. Preference among exploiters is modeled by a 3-layer perceptron with mutable and heritable nodal weights. The highly defended victims are less fit than non-defended victims in the absence of exploiters, but the situation is reversed when exploiter densities are sufficiently high. The partially defended victims are intermediate in both cases. Exploiters discriminate among undefended (highly palatable) and defended (unpalatable) victims by responding to mutable, heritable cues associated with each victim.

The behavior of the model depends on 5 key parameter groups, which determine: (i) the relative fitness of the three victim-defense phenotypes, (ii) how the fitness functions of these phenotypes respond to increasing exploitation rates, (iii) the relative fitness of an exploiter in responding differentially to different mixes of the three victim phenotypes, (iv) ecological competition for both the victim and exploiter populations, and (v) the mutation rates for the victim-cue and exploiter-response phenotypes.

Victim cue-phenotypes (*x, y*) are points in the 2-dimensional signal space 0≤*x*≤1, 0≤*y*≤1. The victims are resources for the exploiters. Each victim belongs to one of three clusters *c* (=1,2,3), where each cluster represents one of the three defense phenotypes described above (highly-, partially-, un-defended). The relative fitness of the victim defense-phenotypes in the absence of exploiters, and in order of vulnerability, are given by *v_c_*, *c*=1,2,3. Victim fitness is also reduced by intra-defense-phenotype competition. We assume spatial segregation of different fixed defensive phenotypes, and thus ignore inter-defense-phenotype competition; although the analysis is easily extended to include the latter. The number of exploiters and victims depend on actual values of fitness functions in each generation and vary between 47–55 (victims) and 8–53 (exploiters) once the cycle has settled in. Specifically, the fitness of victim *j* is a sigmoid function of the size (*P_c_*) of the cluster of which it is a member, its egg-load (*e_j_*), and its resource value to exploiters (*v_c_*):

(1) $$W_{j}^{v}=\frac{\beta }{1+e^{\delta (dP_{c}+k_{1}e_{j}+k_{2}(v_{MAX}-v_{c})-\gamma )}}$$

where individual *j* is a member of cluster c.

Parameter *β* determines maximum fitness, *δ* is a slope parameter, *d* sets the intensity of the density dependence. Parameter γ is a population growth rate parameter. Parameter *v_MAX_* sets the maximum resource value a victim can have. The cost parameter for an egg-load is *k*_1_, and for having a defense against exploiters is *k*_2_. We require *k*_1_>*k*_2_ since the victims trade off predation costs by expending fitness currency on defense.

Individual victims are given a random time for reproduction within the generation span (*g_s_*) of the exploiter. This implies that the exploiters have *g_s_* generations for each victim generation. Victim fitness is then calculated on the instantaneous attack intensity (e.g. egg load) that occurs in proportion to the number of exploiters that respond strongly to the victim. At reproduction, signal mutates with probability *p_s_* for each channel (e.g. chemical cue compound), and its value is modified with a random value between ± *r_s_*.

Exploiters are represented by individual perceptrons (Haykin 1994; Holmgren and Getz 2000) with two input, six hidden, and one output nodes. The fitness *Wi^E^* of exploiter *i* depends on (i) the resource value of the victims it attacks, (ii) the intensity of the attack (e.g. the number of eggs the exploiter lays) on the victims, and (iii) the probability of attack success (e.g. the eggs hatching), and is given by the function

(2) $$W_{i}^{E}=\sum_{j}e_{j,i}\varphi \frac{1}{1+{\left(\frac{e_{j}}{v_{j}{ɛ}_{1/2}}\right)}^{\alpha }}\;\text{where }e_{j}=\sum_{i}e_{j,i}$$

The number *e_j_*,*_i_* of attacks on victim *j*, by exploiter *i*, depends on the exploiter response (Holmgren and Getz 2000). Parameter *φ* is the maximum attack intensity (e.g. the number of eggs each exploiter carries). Here *α* is a parameter that determines the abruptness of the effects of density dependence (Getz 1996). Parameter *ɛ*_1/2_ sets at which value of *e_j_* where the sigmoid fitness function returns half its maximum value. The exploiters have clonal reproduction during which mutations of the synapse strengths of the neural network occur with probability *p_n_*. When they mutate, the synapse strength is modified with a random value between ±*r_n_*. The synapse strengths are limited to [−*s_r_*, *s_r_*]. The reproductive ability of an individual is given by the individual’s fitness plus a random value between 0 and 1. The number of offspring an individual produces is the reproductive ability rounded to the closest integer. All random values are drawn from a rectangular distribution.

### Parameter values

We report simulation results for the set of parameters listed in Table 1, with comments regarding the robustness of the observed behavior to perturbations in the value of these parameters.

The observed behaviors occur over a range of parameter values that support the coexistence of two exploiter phenotypes on the structured victim population. The value of the ecological parameters in our model are set to support sufficient individuals from both victim- and exploiter population to coexist, and that their relative values are ecologically motivated. To be able to distinguish among victim cue-phenotypes the exploiter’s perceptrons is given sufficient hidden units. The evolutionary parameter values are set to keep the evolutionary rates significantly slower than the ecological rates.

The magnitude of victims’ cost for defense is a lively discussed topic (Strauss et al 2002; see Mauricio 1998). The value of *k*_2_ in our model is not directly correlated to the loss of fitness for victim defense. The actual loss of fitness due to the cost for defense in the presence of exploiters depends on the combination of all the cost parameters in the victim fitness function. We use (1) to calculate an example of the fitness loss due to the cost of victim defense. With the parameters given for the model above we calculate the fitness for an average highly defended individual which has a cost for the defense *(k*_2_ = 0.35*)* in a victim cluster of 18 individuals *(P_c_* =18*)* and 18 exploiters all attacking by laying one egg (*e_j_*=1) on each of the victims in the cluster. We compare this fitness to the fitness of an individual in the same system, which lacks the cost for defense (*k*_2_=0). The fitness reduction for having a defense is about 23%. If each exploiter attacks by laying 2 eggs *(e_j_*=2*)* the fitness reduction for having a defense becomes 72%. Comparable levels of fitness loss due to victim defense have been reported in the literature (Strauss et al 2002).

## Results

In an individual-based approach to modeling victim-exploiter interactions, the response of each neural net (i.e. exploiter) to victims located at different points in the signal space is calculated. The response of each neural net at any point in the signal space takes on values either close to zero or to one (Holmgren and Getz 2000). This implies that the average response of all exploiters in any point in the signal space effectively represents the proportion of exploiters in the population that would exploit a victim if its cue phenotype were located at that point.

**Figure 1:**
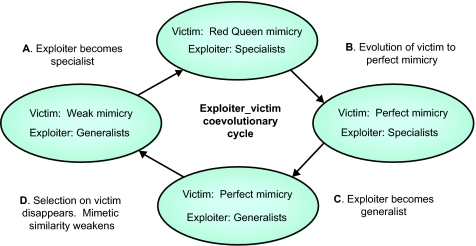
A schematic view of the transitional stages in exploiter-victim systems. (A) With distinguishable victims, the exploiter will diverge into specialists. (B) Exploiter preferences put directional selection on victims: for undefended to become similar to defended, and for defended to be dissimilar to undefended. This results in “red queen mimicry”, driven by the exploiters’ discrimination. We chose the Red Queen concept as a simile for the mimicry dynamics since the dynamics of the system forces the victims to evolve their signals rapidly in the exploiters’ perception landscape without affecting their relative situation since all victim strategies evolve at the same time and so do the perception landscape of the exploiters. (C) Defended and undefended victims become identical. Exploiter can no longer distinguish victims and becomes a generalist. (D) With exploiter generalists, there is no selection gradient on victims. Victims will drift apart as mimicry weakens. The process repeats itself.

Over a range (extend unknown) of victim-cue and exploiter-perceptual phenotypes, the model settles into a continuous cycle of coevolutionary stages (Fig 1).

**Figure 2:**
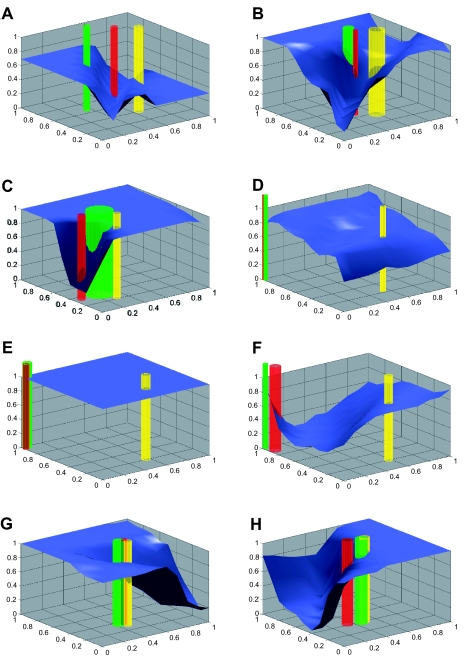
Victim-cue phenotypes and exploiter-response phenotypes are plotted above the 2-D signal space. The two bottom axes represent signal strengths of cue phenotype. The blue surface shows the average response of all exploiters to a test signal at any point in the signal space. The vertical columns represent victim clusters: green—undefended, yellow—intermediate, and red—defended. The tube center is at the average of the victim cue-phenotypes in the cluster in question and the radius is the standard deviation. The images are captured after the following number of generations: (A) 6 000 (B) 20 500 (C) 75 500 (D) 140 000 (E) 148 000 (F) 154 900 (G) 250 000 (H) 277 000. The panels are captured from a video of output from our coevolutionary model. The supplementary video shows the complete video output from this simulation.

Initially, all individuals of the same defense phenotype are located at the same point in the victim-cue phenotype space, with the low defense phenotype lying between the high defense and moderate defense phenotypes. For the first 6000 generations the victim-cue phenotypes do nothing more than acquire some variability around their initial starting values, as represented by the width of the three cylinders in Fig 2A.

**Figure 3:**
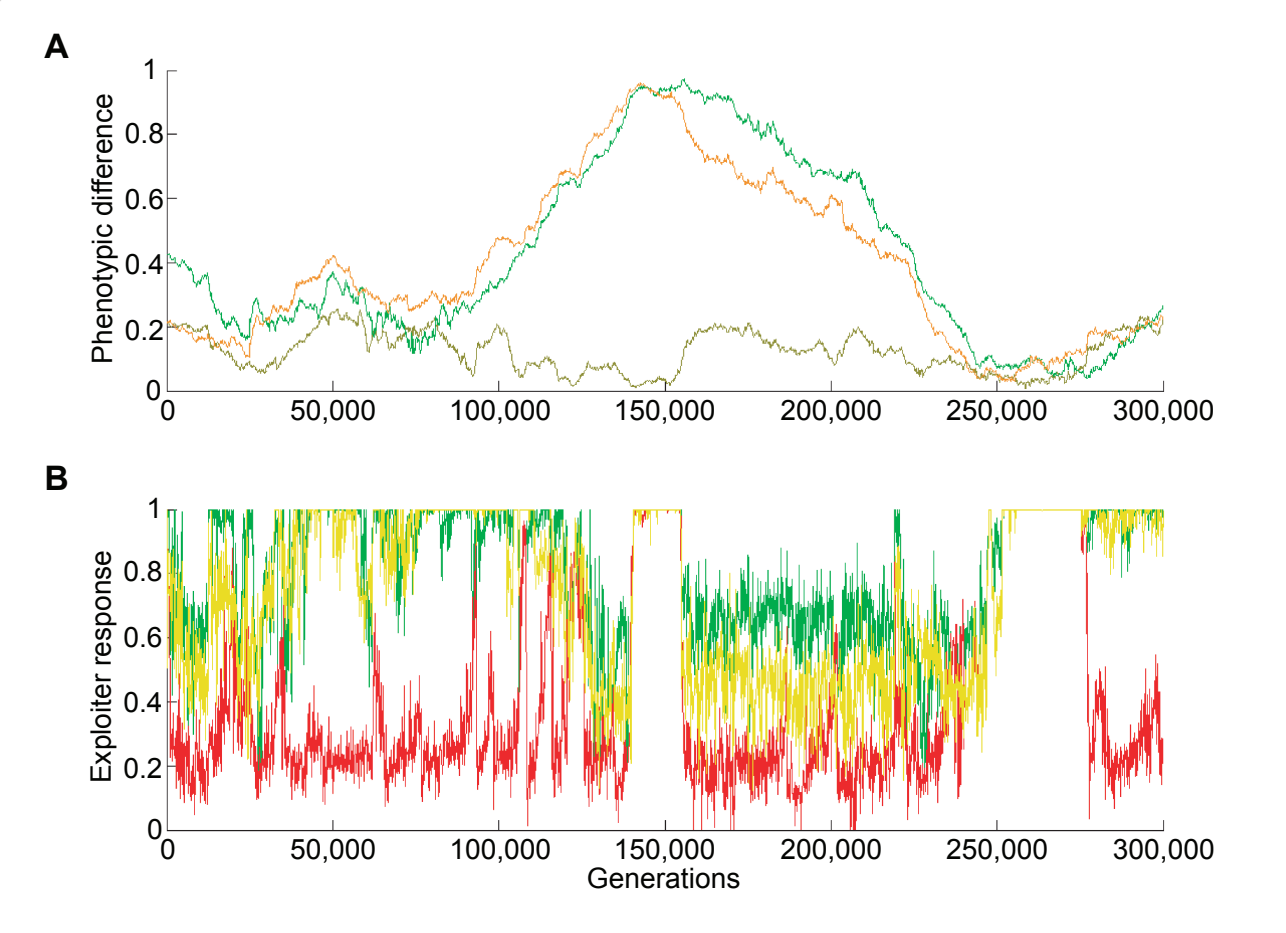
Exploiter responses to victims and Euclidean distances in cue phenotype space between victim clusters over simulation time. (A) The phenotypic difference (represented by Euclidean distance between centers of victim clusters): olive-green line—between most and least defended, yellow-green line—between least and intermediately defended, orange line— between most and intermediately defended. (B) The average exploiter response to the average victim of each defense-phenotype: green line—undefended victims, red line—defended victims, and yellow line—partially defended victims.

The exploiter phenotypes, however, radiate into a three-lineage guild (Fig 3B): generalist exploiter phenotypes on all three types of victims, and two specialists phenotypes, one on each of the two more palatable victims. Laboratory experiments investigating specialization in insects reveal a small number of generalist insects coexisting with more specialized insects (Caillaud and Via 2000). These exploiter lineages are of unequal size, because the system tends towards an ideal-free distribution, i.e. different victim-defense phenotypes have the same fitness after exploitation and competition processes have been taken into account (Holmgren and Getz 2000; Fretwell and Lucas 1970).

The averaged responses of the exploiters over the signal space represent an exploitation landscape that inversely determines the relative fitness of individual victims of the same defense phenotype. Selection acts on victim-defense phenotype populations to move their average cue phenotype down the response surface: that is, in a direction that reduces the proportion of exploiters responding in that part of the cue space. The variance of the victim cue-phenotypes is represented by the radius of the correspondingly colored column protruding through the response landscape (Fig 2). The speed of evolutionary change of average victim cue-phenotype, in the populations of each of the defense types, depends on the mutation rate, the cue variance, and the slope of the surrounding response landscape. Essentially, the progeny of individual victim-cue phenotypes lower down on the response surface are selected in favor of the progeny of those higher up on the response surface. Evolution proceeds by edging the cue phenotypes of the most preferred victims (green, undefended) down the response surface landscape in the direction of the cue phenotypes of the least preferred victims (Fig 2A, B and C), essentially as a Red Queen process: i.e. the model has to continuously evolve to maintain the distance to, and hence the exploiters ability to discriminate it from, the “chasing” mimic (Van Valen 1973; Holmgren and Enquist 1999). Eventually complete mimicry emerges (most defended and least defended phenotypes coincide— Fig 2D, E) once constraints on the evolution of the cue phenotype come into play (cues are bounded below by zero and above by saturation phenomena). During the Red Queen phase, the size of the gap between the Batesian mimic and its model depends on the ability of the exploiters to rapidly evolve the ability to discriminate between similar signals (Getz and Smith 1987). In our model, the degree of success is represented by the height of the perceptual response surface around the victim-cue phenotype clusters. In short, the situation can be envisaged as the un- or partially-defended mimics surfing down a moving response wave in the direction of the defended model phenotypes, with the steepness of the wave maintained by adaptation of the exploiter’s differential response to victim mimics and models (Fig 2C).

Aside from constraints on cues, mimicry can also be complete when the model phenotype gets trapped between mimic phenotypes (Fig 2G; around generation 250,000, cf Fig 3 A). Of course, in higher dimensional signal spaces, the model phenotypes have more room to evade mimics, but can still get trapped or slowed down by constraints on rates of adaptation. Once mimicry is complete, exploiters are no longer able to discriminate between victim-defensive phenotypes. Mutational drift leads to rapid regression of perceptual specialization in exploiters, i.e. the specialized pathways in the brain needed to maintain categorization skills are no longer under constant selection and hence degraded as the system neutrally moves to the higher entropy state of a generalist. If victim mutation rates are not too fast compared with exploiter mutation rates, after some hundreds of generations, exploiters become indiscriminate generalists (Fig 2E—the average response surface has taken on the value 1 everywhere). The coevolutionary process remains directionless for some stochastic period of time, until one or more highly defended victim phenotypes drift or jump (evidence exists for saltational evolution of chemical cues; Symonds and Elgar 2004) to a cue phenotype sufficiently different from the rest to allow selection to begin once more. The expected value of this time depends on both victim and exploiter mutation rates. Once the new direction is set, selection serves to accentuate the initial random fluctuation and the Red Queen process starts anew. The defended phenotypes escape their Batesian mimics (Fig 2F, H) and the exploiters adapt by specializing once more at a rate dependent on mutation and fitness parameter values.

## Discussion

Our results suggest four principal stages of the coevolutionary process in which the victims and the exploiters evolve sequentially (Fig 1): (A) Exploiters evolve into specialists where the degree of specialization is likely to increase with increasing complexity in the number of resources, particularly if generalists do more sampling of resources before deciding to make a meal of any and perceptual acuity is neurally constrained in individual exploiters (Bernays et al 2004; Bernays 2001); (B) With specialists in place, conditions are set up for the evolution of mimicry under a Red Queen process that is finally thwarted by constraints in the production of ever more extreme cues; (C) With the constraints in place, a period of complete mimicry arises and all specialists rapidly revert to generalists; (D) Finally, random drift allows the mimicked population to escape followed soon thereafter by an adaptive response of exploiters towards specialization.

The cyclic properties of our system match a previous study of a coevolutionary chase in an exploiter-victim system where the stabilizing properties and the strength of inter- and intra-species interactions express a cyclic behavior during coevolution (Gavrilets 1997). Even though our systems show a cyclic behavior for different properties, the coevolutionary dynamics of exploiter-victim systems generally seem to express a cyclic property. Different manifestations of the cyclic tendencies of coevolving systems have been reported in previous studies (Abrams and Matsuda 1997; Dieckmann et al 1995).

For the combination of parameters selected, our system spends most of the time in the stage of exploiter specialization (Fig 3B), with differential response to the victims (Fig 2A). For shorter periods, exploiters become complete generalists. The strongest factor affecting the evolution of specialization is the strength of selection acting on the exploiter. Increasing mutation rates of cues increases the turnaround time of the coevolutionary cycle and counteracts higher degrees of specialization. Also, greater levels of specialization are associated with larger differences in defense-phenotypes among victims.

Although models of population genetics and evolutionary processes often have equilibria, the fossil evidence reveals a punctuated process of quasi-equilibria (Gould 2002). Our model reveals the same pattern with long periods of a Red Queen mimicry process ultimately halted by constraints on victim discrimination cues followed by a saltational change of phase from several specialist to one generalist exploiter phenotype (as evidenced by the rapid changes in the response time series at around 140, 154, 245 and 280 thousand generations: see Fig 3B). Further, our model demonstrates that although the coevolution of traits may well be asymmetric over short periods of time, over longer periods in the Red Queen chase symmetry arises in terms of the exploiter and victim continuously trading places as leader and follower.

Two recent papers (Janz et al 2001; Nosil 2002) concur with our view that the evolution of host range is a highly dynamical process not directed solely toward specialization. Although Nosil 2002 found that the transition rate from generalists to specialists generally is significantly higher than the reverse transition rate, they also found high transition rates toward generalization. Our results support these findings with generalization occurring naturally as part of the coevolutionary cycle, but at a lower rate than specialization.

Although a caricature of reality, our model reflects interactions across three trophic levels. In plant-insect interactions cues are typically compounds of the type found in green odor (Hatanaka 1993). The relative ratios of these compounds are known to be discriminable by the insect olfactory system (Dejong and Visser 1988), with the response modeled by a neural net (Getz and Lutz 1999). Similarly, parasitoids are known to cue in on chemical volatiles produced by invertebrate hosts and the plants on which these hosts feed (Vet 2001). Some predators, on the other hand, use characteristic sounds to identify their victims (Pollack and Imaizumi 1999), or vision, as in the classic Batesian mimicry example where butterflies attempt to avoid their avian predators (Devries 1987). The model isn’t developed to simulate a particular two-species interaction. The model is developed to capture the dynamics of a signal recognition mechanism system at three levels; behavior, ecology and evolution. A typical system in which this mechanism is vital is the system where insects choose host plants, as described above. Other plausible systems are parasite-host systems, snail and slug interactions with plants etc.

Johnstone 2002 explains the occurrence of imperfect mimics (mimics with poor similarity to their model) in a system based on kin-selection. One implication of the results from our model is that an imperfect mimic strategy may arise in the victim population due to poor or unfocused discrimination by the exploiters. Victim discrimination is a delicate process during the Red Queen mimicry phase and strong selection pressure acts on exploiters trying to discriminate fully defended victims from undefended victims with very similar cue phenotypes. An exploiter’s fitness loss for not attacking a third victim cluster, peripheral to our two Red Queen mimicry clusters, can be compensated by the fitness gained from an ideal discrimination between these two Red Queen mimicry clusters. This could lead to exploiters focusing their discrimination mechanism on discriminating Red Queen mimics. Evidence of this focus in exploiter evolution is shown in the relatively stable response gradient between mimicking victims while the rest of the response landscape continuously exhibit abrupt changes, as seen in the supplementary video. If the third population of victims is similar enough to the model it can be taken for an imperfect mimic.

A phylogenetic study on cowbirds (Icterinae) has been interpreted as brood parasitism evolving once from an ancestral specialist towards several generalists (Lanyon 1992). Rothstein et al (2002) argues that the results of this study are more consistent with the hypothesis that these cowbirds coevolved from an ancestral generalist cowbird that gave rise to two species that separately evolved a more specialized host strategy. Lanyon (1992) bases the results on parsimony in the evolution of specialization, while Rothstein et al (2002) shows how coevolution, through specialization, can explain the same results. This particular example provides some credence to our analysis, although more studies are needed to assess whether the mimicry cycles predicted by our model really does occur and, if it does, how wide spread it is.

## Conclusion

We demonstrate that reversals in the evolution of specialization can arise quite naturally as part of an asymmetric and four-phase coevolutionary process. Specialization, if linked to assortative mating, may provide conditions for speciation (Dieckmann and Doebeli 1999). Assortative mating can, among other things, be facilited through learning; and learning by itself can accelerate speciation under certain conditions (Lachlan and Servedio 2004). If a hybridization barrier is created during the specialization phase of the cycle, the exploiter specialist lineages will remain as distinct exploiter species in the generalization phase, although being generalists with overlapping niches. If, on the other hand, the specialization phase is too short to generate a hybridization barrier, the exploiter specialist lineages will merge back to one species. Hence, this cycling process in signal-response based exploiter-victim systems may play a role in speciation, the intermittency of which has not previously been discussed. Although the context for our analysis has been exploiter-victim specialization and Batesian mimicry, our modeling approach could be extended to sexual selection, or take into account that defense phenotype of victims can also evolve.

## Figures and Tables

**Table 1:** Parameter values generating the results seen in Fig 2 and 3.

Parameter	Symbol	Base value
Resource value of victim in cluster1	*v_1_*	1.5
Resource value of victim in cluster2	*v_2_*	1
Resource value of victim in cluster3	*v_3_*	0.2
Maximum fitness of victim	*β*	1.1
Slope parameter of victim fitness function	*δ*	4
Intensity of density dependence	*d*	0.6
Population growth rate	*γ*	12.2
Maximum resource value of victim	*v_max_*	1.5
Cost for egg-load	*k_1_*	0.7
Cost for a victim’s defense	*k_2_*	0.35
Generation span of victim population	*g_s_*	50
Signal mutation probability	*p_s_*	0.01
Signal mutation range	*r_s_*	0.05
Maximum attack intensity (available eggs)	*φ*	2
Abruptness of density dependence in exploiters	*α*	3
Half-initial-value constant of exploiter fitness function	*ɛ*_1/2_	1
Probability of synapse strength mutation	*p_n_*	0.1
Range of synapse strength mutation	*r_n_*	0.5
Range of synapse strength values	*s_r_*	10

## Appendix

### Supplementary Video

This movie shows the video output from the complete simulation described in the manuscript. Figure 2 in the manuscript contains selected panels from this simulation. Victim-cue phenotypes and exploiter-response phenotypes are plotted above the 2-D signal space. The two bottom axes represent signal strengths of cue phenotype. The blue surface shows the average response of all exploiters to a test signal at any point in the signal space. The vertical columns represent victim clusters: green— undefended, yellow—intermediate, and red— defended. The tube center is at the average of the victim-cue phenotypes in the cluster in question and the radius is the standard deviation. The simulation covers 300 000 exploiter generations. See the manuscript for more information about the interpretation of the contents of the movie.
